# The Potential of Stereolithography for 3D Printing of Synthetic Trabecular Bone Structures

**DOI:** 10.3390/ma14133712

**Published:** 2021-07-02

**Authors:** Ana Grzeszczak, Susanne Lewin, Olle Eriksson, Johan Kreuger, Cecilia Persson

**Affiliations:** 1 Department of Materials Science and Engineering, Uppsala University, 751 21 Uppsala, Sweden; susanne.lewin@angstrom.uu.se (S.L.); Cecilia.Persson@angstrom.uu.se (C.P.); 2 Department of Medical Cell Biology, Uppsala University, 751 23 Uppsala, Sweden; olle.eriksson@mcb.uu.se (O.E.); johan.kreuger@mcb.uu.se (J.K.)

**Keywords:** stereolithography, trabecular bone, additive manufacturing, 3D printing, mechanical properties

## Abstract

Synthetic bone models are used to train surgeons as well as to test new medical devices. However, currently available models do not accurately mimic the complex structure of trabecular bone, which can provide erroneous results. This study aimed to investigate the suitability of stereolithography (SLA) to produce synthetic trabecular bone. Samples were printed based on synchrotron micro-computed tomography (micro-CT) images of human bone, with scaling factors from 1 to 4.3. Structure replicability was assessed with micro-CT, and mechanical properties were evaluated by compression and screw pull-out tests. The overall geometry was well-replicated at scale 1.8, with a volume difference to the original model of <10%. However, scaling factors below 1.8 gave major print artefacts, and a low accuracy in trabecular thickness distribution. A comparison of the model–print overlap showed printing inaccuracies of ~20% for the 1.8 scale, visible as a loss of smaller details. SLA-printed parts exhibited a higher pull-out strength compared to existing synthetic models (Sawbones ™), and a lower strength compared to cadaveric specimens and fused deposition modelling (FDM)-printed parts in poly (lactic acid). In conclusion, for the same 3D model, SLA enabled higher resolution and printing of smaller scales compared to results reported by FDM.

## 1. Introduction

As reported by statistics on hospital-based care in the United States, the number of orthopaedic procedures concerning all age groups amounted to 3,399,600 in 2005 [1]. Bone models are used by surgeons during preoperative planning of such procedures, and in visual and haptic surgical simulations for training in low-stress environments [2,3]. Bone models are also needed to test and design medical equipment, such as orthopaedic screws and implants. However, the development of synthetic models with high resemblance to natural bone remains challenging due to the unique properties and structure of bone tissue [4]. Although cadaveric specimens may provide the most accurate representation of the mechanical behaviour of bone, reproducible results are difficult to achieve due to differences among individuals, anatomical sites and medical conditions [5]. Synthetic models could provide more consistent morphologies, increase repeatability and be more readily available [6]. With respect to ethical concerns, such models could help reduce the number of animals used in experiments [7]. In addition, synthetic models could help reduce the experimental time and cost by avoiding the need for ethical approval and allowing for faster sample preparation. Furthermore, synthetic samples do not need to be handled under special conditions, such as controlled temperature or immersion in phosphate-buffered saline solution.

The currently available synthetic models do not accurately mimic the inherent highly porous and complex structure of trabecular bone [4], neither in terms of internal structure nor in terms of mechanical properties [5,6]. Poukalova et al. [8] investigated the relationship between trabecular microstructure and mechanical properties (elastic modulus, compressive strength and suture anchor pull-out strength) in both cadaveric specimens and in commonly used synthetic trabecular bone models (Sawbones ™). Several microstructural properties differed between the cadaveric bone and the Sawbones ™, e.g., trabecular thickness. The cadaveric specimens showed a significant correlation between trabecular thickness and elastic modulus, but the same correlation was not found for the Sawbones ™. This result was explained by the limited variability in trabecular thickness, even when using Sawbones ™ with different densities. This showed that the use of commercially available synthetic bone may not be suitable for specific applications and that the bone structure is an important aspect to replicate as it has a significant influence on the mechanical properties. In another study, Patel et al. [6] tested low-density Sawbones ™ mimicking osteoporotic bone and proved these models to be unsuitable to represent osteoporotic cancellous bone in certain aspects, e.g., energy dissipation effects. Sawbones ™ synthetic bone models are produced from polyurethane foam, and the ability to influence the microstructural properties such as trabecular thickness is limited. In response to these limitations, additive manufacturing techniques are emerging as an attractive way of producing the desired bone models [9]. Indeed, the fabrication of biomimetic bone scaffolds has been the object of extensive research over the past two decades, with specific design considerations such as biodegradability, mechanical similarity and control over the pores’ size [10]. Similarly, bioprinting strategies have been extensively investigated to demonstrate the ability to create vascularised cell-laden bone constructs [10]. However, techniques allowing for printed structures with both geometrical accuracy of microstructural features and desired mechanical properties remain to be developed. Yang et al. reported on scaffold fabrication processes for the repair of large segmental bone defects, and especially on the current characteristics of several typical additive manufacturing techniques, their advantages and their limitations [11]. The latter are mainly related to insufficient densification of the printed material, low mechanical strength, low resolution or poor surface quality [11]. Alsheghri et al. [12] encountered challenges comparing mechanical tests results with numerically predicted mechanical properties at small scales due to printing defects, and required printing scaled up geometries. Thus, it appears necessary to investigate printing techniques that allow for high printing resolution. Wu et al. [13] evaluated the possibility of using fused deposition modelling (FDM) to produce trabecular structure. The technique showed potential, but limitations were encountered in printing resolution. Furthermore, they observed an unsuitable effect of the layer-by-layer construction process on mechanical properties.

In a review, Szymczyk-Ziólkowska et al. [14] reported that recent additive manufacturing technologies can enable the precise manufacturing of porous scaffolds, yet this is restricted by the resolution achievable with the different available materials. Ng et al. [15] and Li et al. [16] performed reviews on the potential of vat-polymerisation printing techniques (stereolithography (SLA), digital light processing, two-photon polymerisation) for bioprinting and tissue engineering applications. They highlighted that the vat-polymerisation printing techniques allow for a superior resolution as compared to other 3D printing methods. However, despite very promising recent studies, the highly complex bone structure has not yet been accurately replicated with bioprinting techniques [15]. Chartrain et al. [17] also showed that the vat-polymerisation techniques allow for repeatability and precision, thus presenting a significant advantage over traditional scaffold fabrication techniques, even if the resolution is often achieved at the expense of the size of the part that can be printed. Other techniques, for example solvent casting or particulate leaching, present drawbacks such as the inability to control pore shape and pore interconnectivity [17]. Vat-polymerisation appears promising, but only simple architectures have been investigated with the most precise printers [17]. Mézière et al. [18] used SLA, which is a 3D printing method using a photo-reactive, resin-solidified, point-by-point by a focused laser beam, to print horse trabecular bone models and perform ultrasound measurements. The models were printed with reasonable accuracy, but their mechanical properties were different from those of real bone. Barak and Black [19] used a feeding method consisting of a spray of droplets of photopolymerizable material in a Poly-Jet device coupled with a model reconstructed from micro-CT scans of chimpanzee bone, to study changes in bone morphology and the effect of trabecular thickness on mechanical properties. This model was chosen because chimpanzees have thicker trabeculae compared to humans and a larger thickness was preferable, given the limitations in printing resolution. Printing equipment and materials allowing for a higher resolution remain to be assessed (Table 1).

This study aimed to evaluate SLA as a means of producing structures replicating human trabecular bone, including a volumetric comparison with the model for a highly detailed assessment of the printed parts, with the following objectives:

- To acquire data on the suitability of SLA technology for the intended application.
- To evaluate replicability in trabecular spacing, thickness and total porosity, with micro-computed tomography (micro-CT) analysis.
- To evaluate mechanical properties with compression testing and screw pull-out tests.

## 2. Materials and Methods

### 2.1. SLA Printing

The 3D bone model used for this project was generated during a previous study, and the process has been described in detail elsewhere [13]. A short summary is provided here for clarity and context. The 3D images were obtained through synchrotron micro-CT scanning of a human femoral head sample. The raw images had a resolution of 3.25 µm. A series of image processing steps was applied for segmentation of only the bony part. Unconnected debris resulting from bone cutting were removed. Finally, the segmented bone was saved as a printable stl file. A 3D mesh of a cylindrical region of interest was generated with MATLAB (Natick, MA, USA). Due to the size and large number of images, and the associated computational burden, the processing steps resulted in a reduction in resolution by a total factor of 8, with the voxel size being ultimately equal to 26 µm.

The 3D model of the bone was imported into the slicing software Preform (Formlabs, Somerville, MA, USA). 3D printing was performed on Form2 and Form3 (Formlabs, Somerville, MA, USA) SLA printers. The printed material is a resin made out of a thermoset polymer, composed of a mixture of methacrylic acid esters, photo-initiators, proprietary pigment and additive package. It contains 75% to 90% of methacrylated oligomer, 25% to 50% of methacrylated monomer and traces of diphenyl (2,4,6-trimethylbenzoyl) phosphine oxide (<1%) [22]. Commercially available resins have different characteristics and potential, as specified by the manufacturer, and some of the properties of Formlabs’ resins can be found in Table 2.

Once the printing process was finished, the parts were rinsed in isopropyl alcohol (IPA), which dissolves the remaining uncured resin of the surfaces of the part. Then, the parts were post-cured using UV-light to improve and stabilise the mechanical properties, in accordance with the manufacturer’s recommendations (Table 3). UV-light exposure triggers the formation of additional chemical bonds in the cured resin and makes the part stronger and stiffer, while heat exposure accelerates the process of bond formation [23,24].

Available resins from Formlabs were used without changing their composition. The Black resin, which is among the standard resins, was expected to provide the best results in terms of structural accuracy and printed details due to its opacity preventing the laser light from penetrating deeply into the resin, and thus preventing artefact formation due to over-cured resin. Its fluidity also allows the liquid resin to properly flow out of the part during the printing process. To evaluate the SLA printer capability, the three-dimensional trabecular bone model previously mentioned was used as an input model in a Form3 printer. This model was scaled up in Preform with the following scaling factors: 1 (original size), 1.3, 1.5, 1.8, 2, 2.3, 2.5, 2.8, 3, 4 and 4.3. The prints were configured with a resolution of 25 µm and the addition of external support structures that were required due to the overhanging parts of the model.

To assess the mechanical properties of the printed material, standard dense cylinders with a diameter of 6 mm and a height of 12 mm were printed, following ISO 5833 Standard [25] and allowing for standard compression testing.

### 2.2. Mechanical Testing

#### 2.2.1. Compression Tests

Compression tests were conducted on a Shimadzu AGS-X universal testing machine (Shimadzu, Kyoto, Japan), associated with the complementing software package TrapeziumX (Shimadzu, Kyoto, Japan). The displacement measurement was provided by the crosshead position. The compliance was measured as the inverse of the setup stiffness and was corrected in the displacement data for the final results. The load cell had a capacity of 5 kN. The same setup was used in all mechanical testing.

Uniaxial compression tests were performed as preliminary tests on cylindrical dense specimens (6 mm in diameter and 12 mm in height) printed in Black resin, at a crosshead speed of 1 mm/min. The data collected before the force reached 2 N were removed. The stiffness of the samples was defined as the slope of the straight line that fits the force–stroke curve in the linear region, which was delineated independently for each curve. The machine compliance value was used to correct the stiffness, and then the elastic modulus was calculated. These preliminary tests (details are provided in the Supplementary Material, Table S1, Figure S1) followed an experimental plan to test and choose the printing parameters. Each batch included 5 samples. The influence of the following parameters on the mechanical properties was investigated: printer type, orientation, resolution, UV-curing time and preload during the test. The parameters of the control batch were defined as the parameters recommended by the supplier. The expected elastic modulus for the control group was 2.8 GPa (Table 2), but the test result was significantly lower (1879 ± 51 MPa). This elastic modulus is lower than the elastic modulus range of values for cortical bone, as described in Table 1 (12–18 GPa [21]). The elastic modulus of the control group did not appear to be different from the ones of the other batches, except for the batches of the horizontally printed samples. However, in trabecular samples, the initial orientation of the sample may be less important than in dense structures considering that the trabecular structures are oriented in different directions. Finally, it was concluded that the mechanical properties do not seem to be adjustable with the print settings.

Another set of preliminary tests (accessible in the Supplementary Material, Figure S2) using the same equipment and procedure was conducted to assess the influence of post-treatment on the SLA-printed parts. Standard dense cylinders (6 mm in diameter and 12 mm in height) were printed in GreyPro and Durable resin, and only half of the samples underwent post-processing. UV-light and heat exposures were defined according to the supplier’s recommendations. The results showed that the post-treatment greatly increases the stiffness of the parts. However, the elastic moduli obtained with these compression tests were lower than the expected elastic moduli, as provided by the supplier Formlabs (Table 2). The manipulation of untreated samples proved difficult and implied constraints that do not seem acceptable for the intended use of these materials. We decided to use treated specimens as they are more stable.

Uniaxial compression tests were also performed on trabecular samples printed in Black resin at the scales 1.8, 2, 4 and 4.3, and on samples printed at the scale 4.3 that underwent micro-CT. Each batch included 4 samples, except for the unscanned specimens at the scale 4.3 which included 3 samples. As for the standard cylinders, the crosshead speed was 1 mm/min and the stiffness of the samples was defined as the slope of the straight line that fits the force–stroke curve in the linear region, which was delineated independently for each curve. The machine compliance value was used to correct the stiffness, and then the elastic modulus was calculated.

#### 2.2.2. Pull-Out Tests

**Figure 1:**
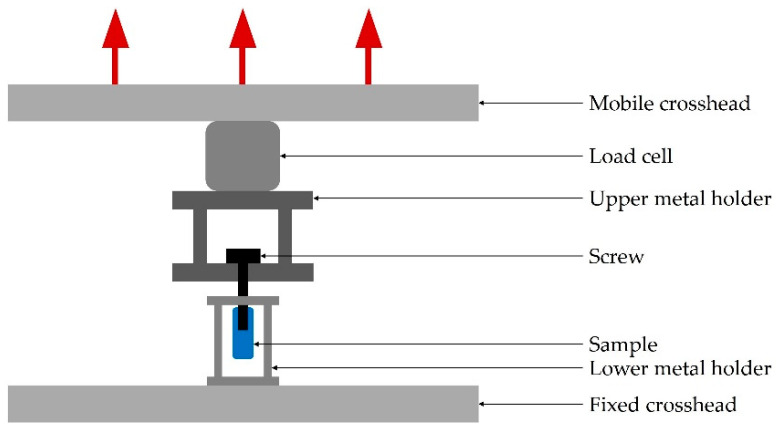
A cross-sectional illustration of the pull-out setup. The loading direction is indicated by red arrows.

The same testing machine was used for pull-out tests, with a crosshead speed of 1 mm/min and a 5 kN load cell. The 6 tested samples were printed with Black resin on a Form3 printer, with a scale factor of 4.3, a resolution of 25 microns and the addition of external support structures. This scale factor was chosen to allow comparison with data from previous studies using this factor [13]. An orthopaedic screw with the following characteristics was used: 6.7 mm outer diameter, 3.5 mm inner diameter, 3.6 mm thread length, 9 mm overall length and 2.8 mm pitch distance. Prior to screw insertion, a pilot hole of 3.5 mm diameter was drilled in the trabecular samples. This operation was performed with machine drilling to ensure correct positioning and fixation of the samples during drilling. The screw was then inserted manually with an insertion depth of 20 mm. The screw was mounted on a steel holder that was attached to the load cell, which in turn was attached to the mobile crosshead. The sample was held by another steel holder, attached to the fixed crosshead. A diagram of the setup can be seen in Figure 1.

#### 2.2.3. Statistical Analysis

Statistical analysis was conducted using SPSS Statistics (IBM, Armonk, NY, USA). The test results are presented as mean and standard deviation. Levene’s test was used to assess homogeneity of variances. If Levene’s test was non-significant, an analysis of variance (ANOVA) was conducted to analyse the differences among the group means. If the ANOVA was significant, Scheffe’s post-hoc test was carried out. On the other hand, if Levene’s test was found significant, Welch’s test was conducted to analyse the differences among group means. Then, Tamhane’s post-hoc test was carried out, to analyse the differences between pairs of groups. The results were considered significant if *p* < 0.05.

### 2.3. Morphological Evaluation by Micro-CT

3D evaluation of the printed samples was performed using a micro-CT scanner, SkyScan 1172 (Bruker, Billerica, MA, USA). The samples were scanned with a voxel size of 12 µm using a voltage of 60 kV and a current of 167 µA. An exposure time of 1100 ms and a rotation step of 0.4° were also used. The same settings were used for all the printed samples, to allow for an unbiased comparison of the results between samples of different dimensions. A different set of parameters was only used to scan the parts that were printed at the original scale. Due to the smaller size of these samples, an increased resolution was necessary to be able to identify internal structures. The chosen settings for these small parts were a voxel size of 5 µm, a voltage of 60 kV, a current of 167 µA, an exposure time of 760 ms and a rotation step of 0.4°. Samples at the original scale printed in all different resins were compared. The purpose was to determine the accuracy that could be reached for each resin.

Due to time limitations, only one specimen of each scale was scanned, except for those that were to be studied in more detail. The list of the scanned samples can be found in Table 4. Visual assessment revealed that the accuracy of the trabecular structures declined for scaling factors below 2, and the small structures were most likely to exhibit errors during printing, so it was decided to scan two samples of the scales 1.3, 1.5 and 1.8, to have more representative results for these scales. Four samples with the scale factor 4.3 were scanned, with this scale being the one chosen during previous studies using FDM [13], and having more representative results on this scale allowed for data comparison.

After scanning, the images were reconstructed using NRecon (Bruker, Billerica, MA, USA) and analysed using CTAn (Bruker, Billerica, Massachusetts, USA). The images were pre-processed by a median filter in order to remove noise. Then, the printed material was segmented using an automated Ridler and Calvard process [26]. Following the segmentation, parts that were not connected were removed and a morphological opening was applied to remove noise. Quantitative analysis was conducted on the segmented volumes in terms of the ratio bone volume/total volume (BV/TV) and trabecular thickness distribution. Trabecular thickness was calculated with 3D analysis in CTAn, using the “sphere-fitting” method by Hildebrand and Rüegsegger [27], i.e., the thickness is set as the diameter of the largest sphere that fits a solid trabecula. The trabecular thickness values were extracted as ranges, with mid-range values and the associated volume of trabeculae (in µm³ or in %) that are measured in this range.

A volumetric comparison of the micro-CT images was also conducted using 3D Slicer 4.10 [28]. The binary micro-CT image dataset which had been used to create the 3D-model of the bone was imported, together with the binary micro-CT image dataset of the printed samples. Datasets from three printed volumes were imported: scale 4.3, scale 1.8 and scale 1.5. For a better comparison, the model was scaled to fit the scale of the printed samples. A landmark-based rigid registration was made to overlap the images of the model and the micro-CT images of the printed samples, and a cylindrical region of interest was created through the volumes (diameter 40 mm). The total volume of printed material was then quantified in the region of interest.

After registration, the volumes of the scaled model and print overlapped. The differences between the model and each print could be observed in more detail after registration, and, more specifically, if material represented in the original model was missing in the printed part (hereinafter referred to as underfilling), and if material present in the printed part was not present in the model (hereinafter referred to as overfilling). To quantify the over- and under-filling, the volume from the model was subtracted from each print (overfilling), and conversely, the volume from each print was subtracted from the model (underfilling). In addition, overlapping 3D models were created to allow for visual comparison of the overfilling.

## 3. Results and Discussion

### 3.1. Mechanical Testing

#### 3.1.1. Compression Tests

**Figure 2:**
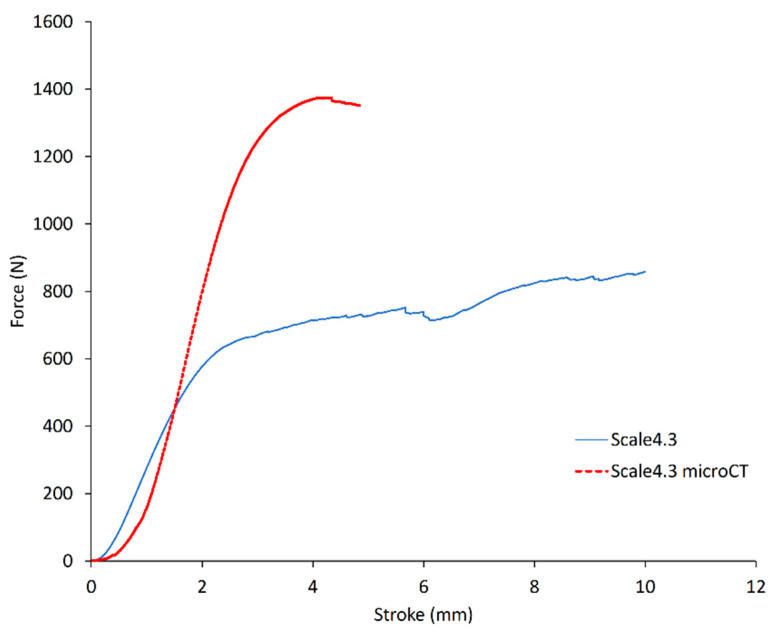
Compression tests of two trabecular samples, representative of their respective batches. One sample (red curve) went through micro-CT before compression. Both were printed in Black resin at the scale 4.3.

Compression tests were conducted on trabecular samples scaled up with different factors, printed in Black resin, and the resulting elastic moduli were measured. Porosity was not accounted for in the calculation of the elastic modulus from the stiffness; instead, an apparent elastic modulus was obtained. The batches did not include enough samples to draw conclusions on any variation of the apparent elastic modulus with the scale factors. However, a significant difference in elastic modulus was observed between samples that were scanned or not. The average elastic modulus for samples printed at the scale 4.3 was 54.11 ± 1.92 MPa, and the average elastic modulus for samples printed at the scale 4.3 that underwent micro-CT scanning was 106.46 ± 13.06 MPa. Thus, micro-CT scanning significantly increases the elastic modulus of the parts. A typical test curve of a 4.3 scale sample and one of a scanned 4.3 scale sample are shown in Figure 2. These curves were chosen as representative of their respective batches. The two types of samples exhibited a very different behaviour during the compression test. This can be explained from the X-ray exposure during micro-CT. One hypothesis is that X-ray irradiation causes the formation of cross-links but also breaks bonds, thus changing the properties of the material, which becomes brittle [29,30]. This phenomenon will have to be considered for possible future studies, in order to avoid altered results.

Tests on standard dense cylinders showed that, for a specific resin, mechanical properties cannot be efficiently controlled by printing and post-treatment parameters. The main resin used for this project was the Black resin from Formlabs. Its composition was not modified, and it was used as recommended by the supplier. The control group, that was composed of samples processed according to the supplier’s recommendations, exhibited an elastic modulus of 1879 ± 51 MPa. The values exhibited by the other groups are available in the Supplementary Material (Figure S1). In future studies, investigations on the influence of resin composition would be of interest. Indeed, it would be advantageous to be able to influence the mechanical properties of the samples by changing the composition of the resin, and thus obtain properties closer to the ones of bone (Table 1). Furthermore, a biocompatible resin could be investigated and evaluated with regard to cell compatibility, which would be a milestone towards in vivo application as bone void-filling scaffolds.

#### 3.1.2. Pull-out Tests

Pull-out tests were performed on six trabecular samples, printed in Black resin at the scale 4.3. Results from a previous study [13] are also displayed in Table 5. Although the stiffness of the SLA samples seems lower than the stiffness of the samples printed by FDM, the FDM results showed large variations within the tested batches, and no statistically significant difference was found.

A list of different studies and their respective maximum pull-out force are displayed in Table 6. The pull-out strength values were scaled by Wu et al. [13] to adapt them to a screw diameter of 6.5 mm and an insertion depth of 20 mm. A linear relation between the pull-out load, screw diameter and insertion depth was assumed [31]. The screw diameter used in the present study (6.7 mm) was very close to 6.5 mm, so it was assumed that scaling was not required.

Shea et al. [32] tested pull-out in Sawbones ™ with a density of 0.08 g/cm³. This foam appears to exhibit a lower pull-out strength than the models tested in the present study. As can be seen in Table 5, the pull-out strength obtained with the SLA-printed trabecular models of the present study is lower than the results obtained for cadaveric samples representing the same bone location (femoral head). It is important to mention that variations could be caused by the pre-mortal condition of the cadaveric specimens. In a study, Palmer et al. [35] performed pull-out tests on Sawbones ™ samples, using a similar setup. The pull-out was conducted with 3.2 drill bit, 6.5 mm screw diameter and 20 mm insertion in 40 mm tall samples. The foam supposed to mimic healthy bone (0.24 g cm^−3^) was used as a control, and the maximum pull-out force achieved was 40 N. This is significantly lower than the pull-out force obtained for SLA-printed parts in the present study: 755.6 ± 146.8 N. Wu et al. [13] conducted a study with models printed in FDM and based on the same input 3D model as the one used in the present study. The pull-out strength achieved (1400–1900 N) was found to be higher than the value found in the current project. In another study, Wu et al. [34] conducted pull-out tests on human femoral head with an average BT/TV ratio of 27.6 ± 6.1, and obtained significantly higher pull-out strength compared to the present study (BV/TV ratio estimated to be 39.5%). To conclude, the values acquired on SLA-printed samples in the present study were higher than previous results obtained with Sawbones ™. The results were also lower than previous results obtained with cadaveric specimens and FDM-printed samples.

In the present study, pull-out was only tested for one group of samples (scale 4.3). Other scaling factors and screws sizes could be tested in future studies. Further pull-out tests could be conducted for samples at different scales, as only samples at the scale 4.3 were tested in pull-out in the present study. All the trabecular samples were printed from the same 3D model. It would be interesting to print samples from another model, to evaluate the variability of the results in printing accuracy and mechanical tests. In addition, the pull-out test setup is challenging in terms of screw insertion, which needs to be fully central and vertical. Any deviation in the insertion technique can bias the results, and this can be a contributing factor to the variability in the results.

### 3.2. Morphological Evaluation by Micro-CT

#### 3.2.1. Bone Fraction Measured with Micro-CT

**Figure 3:**
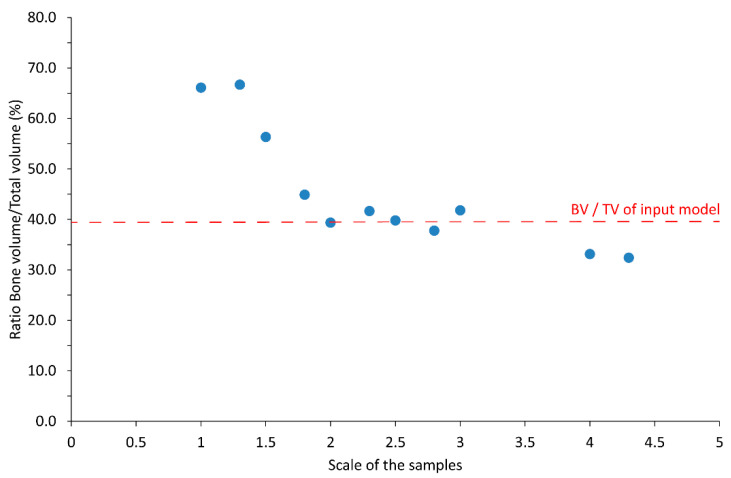
Bone fraction BV/TV (%) as provided after micro-CT and 3D analysis, for samples printed in Black resin at different scales.

The ratio BV/TV was obtained with a 3D analysis in CTAn on the micro-CT images of the samples. The results are displayed in Figure 3. BV/TV was significantly higher for the small scales (66.1% for scale 1, 66.7% for scale 1.3) than for the bigger scales (33.1% for scale 4, 32.4% for scale 4.3). In other terms, the small samples had a much lower porosity. A decreasing trend is visible, i.e., the BV/TV ratio seems to decrease with the increase of the scale factor (Figure 3).

The BV/TV ratio of the input model was estimated to be 39.5%. Prior to conducting the measures, it was assumed that the samples with the bigger scale factor (4.3) would be the best printed samples. Indeed, with the high resolution of the SLA printer, it was expected that big structures should be accurately printed, while some artefacts could appear in the printing of very small features. In fact, for the scales under 1.8, the BV/TV ratio was much higher, and significant artefacts are likely present in the printed samples. This was further investigated by studying the trabecular thickness distribution and conducting a volumetric comparison, as reported in Section 3.2.2 and Section 3.2.3.

It is important to mention that porosity is an overall measure that does not provide details on the location of structures in the sample. It was therefore interesting to study other parameters to compare the printed samples, for example the trabecular thickness distribution, as described in the following section. 

#### 3.2.2. Trabecular Thickness Distribution

**Figure 4:**
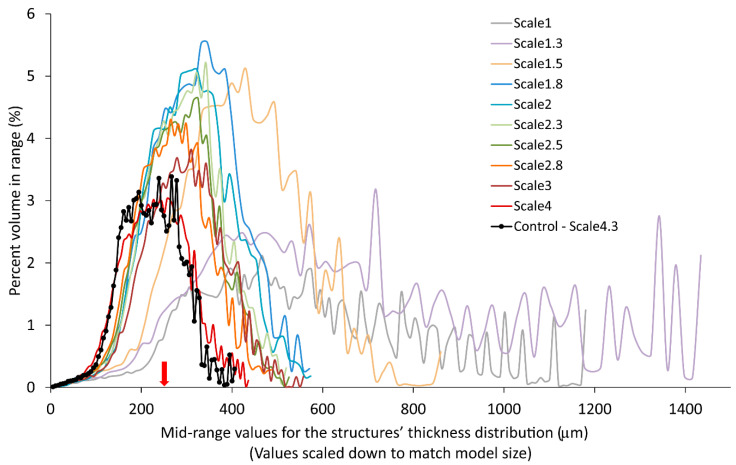
Trabecular thickness distributions for trabecular samples printed in Black resin. The mean for the distribution of the trabecular thickness in the 3D model is 246 µm (marked by a red arrow) [13].

The trabecular thickness distribution is shown in Figure 4. To allow for a comparison between the different scales, mid-range values were scaled down by the scaling factor of their respective samples, to match the original size. Each sample’s trabecular thickness distribution was plotted in a histogram and represented as the percent volume in each range depending on the scaled down mid-range values. However, as the objective was to overlay the distribution from the different samples, the histograms were transformed into curves for better readability. The overlapped distribution curves for the different samples can be seen in Figure 4. The trabecular thickness distribution of the input model was not available, so the sample printed at scale 4.3 was taken as the control. For the control group, the distribution had approximately the shape of a bell curve. This silhouette was also found for samples with scales greater than or equal to 1.8. The curves appear similar but shift and grow, following the decrease in scale factor. For the 1.5 scale distribution, the curve was very shifted and became wider. For the 1.3 and 1 (original) scales, the curves looked very different from the control group. The values were no longer grouped together as in a bell curve but were distributed with a lot of variation. Notably, these samples had fewer thin but also much thicker trabecular structures. This is most likely due to samples with the scale factors below 1.8 containing printing inaccuracies. From this evaluation, it could be concluded that scales 1.8 or 2 are candidates to be the smallest scale for printing accurate structures with this 3D model.

#### 3.2.3. Volumetric Comparison

A volumetric comparison was conducted to quantify the overall volumetric difference between the input model and the printed parts (Table 7). The overall volumetric difference for the scale 1.5 is large (53.8%), but the difference for the scale 1.8 is deemed acceptable (8.9%, i.e., <20%). In fact, the volume difference for the scale 4.3 is not much smaller than the one for scale 1.8, while the scale factor is much larger. There seems to be a scale limit below which the printing accuracy abruptly degrades, and with this analysis the limit appears to be 1.8.

**Figure 5:**
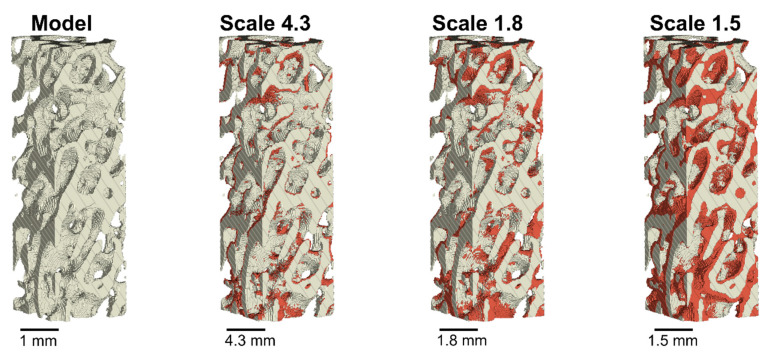
Volumetric comparison of the original bone model and the overfilling volumes from the printed samples at the different scales. The original model is displayed in grey, and the overfilled material in red. Note, the model has been scaled up to the scale of the printed parts in these comparisons.

The difference in volumes was afterwards assessed in more detail by overlapping a scaled version of the model with the printed parts. First, the model volume was subtracted from the printed volume, to obtain the volume percentage of the printed part that was not displayed in the original model (overfilling). Then, the printed volume was subtracted from the model volume, to obtain the volume percentage of the model that was not printed (underfilling). The quantitative results can be seen in Table 8, and a visual representation of the overfilling in Figure 5. Scales 1.8 and 4.3 again appeared to provide acceptable results. As expected, the quantity of underfilling and overfilling was larger for scale 1.8 (23% and 22%) than for scale 4.3 (11% and 15%). However, both scales had a similar amount of underfilling and overfilling, which resulted in a small difference in overall volume compared to the model (Table 7). The comparison for scale 1.5 displayed a large overfilling, as expected according to the results in Table 7. Nevertheless, scale 1.5 instead showed a very low value for the underfilling (5%). This can be explained by the fact that if the sample is very filled, the structures that were in the original model will probably be represented in the printed model, but this may be due to artefacts. For example, if a comparison was made with a sample consisting of a completely dense cylinder, the underfilling would be 0%. However, the high value for overfilling (59%) confirms that a lot of extra material was printed for scale 1.5.

**Figure 6:**
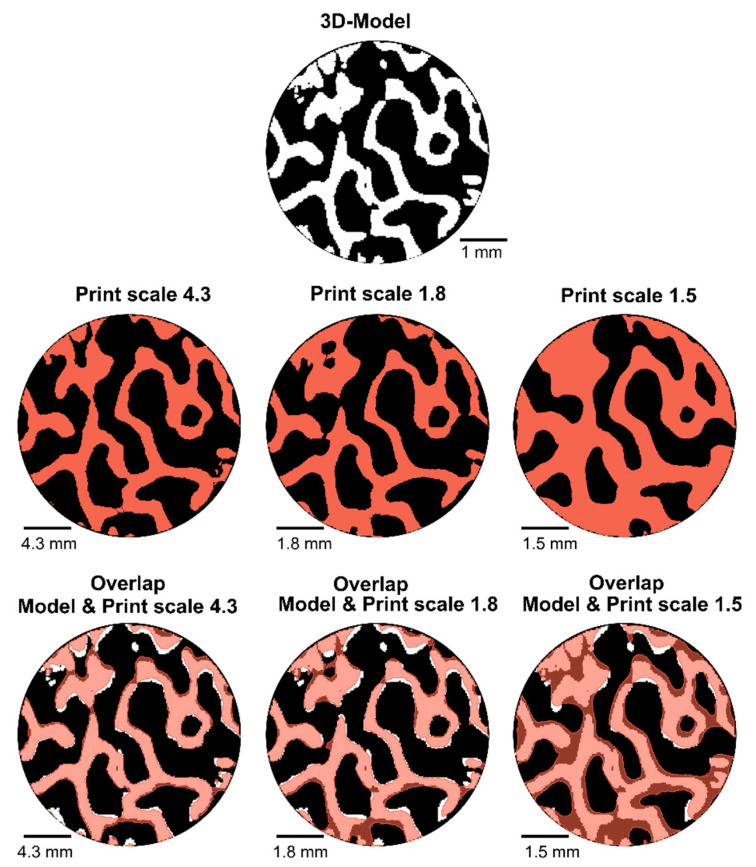
Cross-sectional comparison of registered micro-CT images from the model and the printed parts. The model is displayed in white (**first row**) and the printed parts of different scales are displayed in red (**second row**). An overlap comparison is seen in the bottom rows, where the printed cross-sections are superimposed over the model. For this comparison, the model (**white**) was scaled up to the scale of the displayed printed part (**red**).

A more detailed visual comparison of the overfilling and underfilling could be made in the overlapped cross-sectional micro-CT images of the 3D model and the printed samples. Overlapping images can be seen in Figure 6. The structures of the sample in scale 4.3 appear to be accurately printed, as seen in the resulting overlap images. For scale 1.8, some trabecular structures are too thick compared to the ones of the model, and some anomalies are visible. For scale 1.5 on the other hand, many trabecular structures appear too thick, with the result that some pores disappear and the overall structure is quite different from that of the model. Scale 1.8 appears to be the smallest scale at which it is possible to print the model with acceptable quality and accuracy. However, some errors are noticeable in the printed samples and it could be interesting to conduct further studies on this aspect, to investigate the influence of these artefacts in more detail.

**Figure 7:**
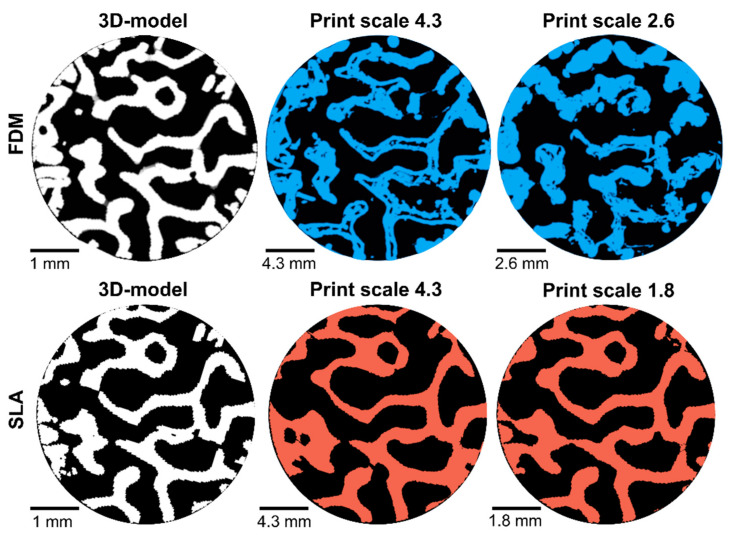
Cross-sectional comparison of registered micro-CT images, of the same part of the 3D-model, from the model and the printed parts produced by FDM (**upper row**) and SLA (**lower row**). Cross-sections for the 3D-models are displayed in white, and the printed parts in blue (FDM) or red (SLA). The images in the upper row are adapted with modifications from [13].

A previous study printed the same trabecular bone model, but with FDM [13]. A comparison of similar cross-sections of the same part of the model printed by FDM and SLA can be seen in Figure 7. These cross-sections highlight the possible underfilling issues encountered with FDM-printing, and the better resolution that can be achieved with SLA-printing. Already at scale 2.6, the trabecular bone model is not printed well by FDM. Interestingly, the difference in total volume between the model and print was less for FDM (0.6%) than for SLA (6%) for samples of scale 4.3. However, it can be clearly seen that in a more detailed analysis, the FDM parts have both more underfilling and overfilling than the SLA part. Overfilling and underfilling was not quantified for the FDM parts, but the trabecular thickness was quantified. Here, the results for SLA (206 ± 117 µm) are closer to the model (246 ± 70 µm) than the results for FDM (98 ± 36 µm). This observation highlights the importance of not only comparing overall volumes, but also doing more detailed observations when evaluating the accuracy of additively manufactured parts. In the evaluation of the trabecular bone model, quantification of trabecular thickness and comparison of over- and under-filling provided a useful representation of the print accuracy.

The results provided by the different evaluation methods used in this study, including the extensive comparison between the model and the printed samples, allowed for a detailed picture of the capabilities of SLA printers and an effective performance comparison with the FDM printing technique. The Black resin permitted to reach a good printing resolution and future studies could focus on modifying its composition to adjust the mechanical properties of the printed parts. Other resins could be investigated, especially biocompatible resin if in vivo applications such as bone void-filling scaffolds are considered. The SLA printing technique proved to be a potential solution to produce anatomical bone models and for training bone models, and in a second instance, aimed to contribute to the overall objective to improve the production of customised medical products.

## 4. Conclusions

Trabecular samples were successfully printed by SLA using a 3D trabecular bone model scaled by factors from 1 to 4.3. Black resin (Formlabs, Somerville, MA, USA) appeared to allow for the best printing accuracy, and the scale 1.8 appeared to be the smallest scale with which to print with acceptable precision. Micro-CT analysis showed that major artefacts may appear in samples with scale factors below 1.8. Trabecular thickness analysis also showed a decreased accuracy in thickness distribution for scale factors below 1.8. A comparison with the total trabecular volume of the model found a difference of less than 10% for samples with scales 1.8 and 4.3, and more than 50% for samples with scale 1.5. Nevertheless, in a detailed analysis of overlapped print and model micro-CT images, scale 1.8 had a loss of accuracy in some details (~20% over- and under-filling). Overfilling was the main problem for the 1.5 scale. 

Compression tests on trabecular samples revealed that X-rays from the micro-CT significantly increased the elastic modulus. Pull-out tests on trabecular samples showed that SLA-printed parts exhibit a higher pull-out strength compared to Sawbones ™, and a lower pull-out strength compared to cadaveric specimens or FDM-printed poly(lactic acid) (PLA) parts. However, for the same 3D model, smaller scales could be printed with good geometric accuracy, and a higher resolution was achieved compared to FDM prints. SLA printing appears promising for the intended application, but further optimisation is required to make 3D-printed structures competitive to the commercially existing foams. 

## Figures and Tables

**Table 1:** Mechanical properties of cortical and trabecular bone.

Bone Type	Porosity (%)	Compressive Strength (MPa)	Elastic Modulus (GPa)
	Fernandez-Cervantes et al. [20]	Milazzo et al. [21]	Milazzo et al. [21]
Cortical	<10	130–180	12–18
Trabecular	50–90	4–12	0.1–0.5

**Table 2:** Elastic modulus for parts printed with Formlabs resin [23]. “Green” parts refer to parts that have not been through any post-processing treatment. “Cured” parts refer to parts that have been through the suggested best post-process treatment (UV-light and heat).

Formlabs Resin	Elastic Modulus for “Green” Parts (GPa)	Elastic Modulus for “Cured” Parts (GPa)
Black	1.6	2.8
GreyPro	1.4	2.6
Durable	0.45	1.26

**Table 3:** Recommended UV-cure for parts printed with Black, GreyPro and Durable resins (Formlabs, Somerville, MA, USA).

Resin	Recommended Cure Time (min)	Recommended Cure Temperature (°C)
Black	30	60
GreyPro	15	80
Durable	60	60

**Table 4:** List of samples analysed by micro-CT. The samples were printed vertically, unless otherwise indicated.

Resin	Scale Factor	Number of Specimens Scanned
Black	1	1
	1.3	2
	1.5	2
	1.8	2
	2	1
	2.3	1
	2.5	1
	2.8	1
	3	1
	4	1 horizontally printed 1 vertically printed
	4.3	4

**Table 5:** Pull-out test results comparison between present study (screw diameter of 6.7 mm) and previous study (screw diameter of 6.5 mm), using the same input model, the same scale factor (4.3) and the same type of screw, with an insertion depth of 20 mm. The same test setup was used for both fused deposition modelling (FDM) and stereolithography (SLA) printed samples. The mean values and standard deviations are presented.

Study	Samples	Stiffness (N/mm)	Pull-Out Strength (N)
Present study Printed by SLA	Black resin	819.3 ± 127.8	755.6 ± 146.8
Wu et al. [13] Printed by FDM	Pure poly(lactic acid) (PLA)	931.1 ± 82.3	1369.7 ± 32.9
	PLA + 5% hydroxyapatite (HA)	1295.2 ± 283.3	1657 ± 446.9
	PLA + 10% HA	1374.7 ± 140.1	1891.3 ± 223.8
	PLA + 15% HA	1374.6 ± 514.8	1555 ± 613.3

**Table 6:** Comparison of pull-out results from the literature.

Study	Material	Insertion Depth (mm)	Screw Diameter (mm)	Pull-out Strength (N)	Scaled Pull-Out Strength (N)
Present study	SLA-printed resin	20	6.7	755.6 ± 146.8	755.6 ± 146.8
Shea et al., 2015 [32]	Sawbones ™, density 0.08 g/cm³	45	6.5	393 ± 9	147 ± 4
Mueller et al., 2013 [33]	Sawbones ™, density 0.16–0.32 g/cm³	22	12.5	700–2500	305–1127
Mueller et al., 2013 [33]	Human femoral head (screw pushed inwards in the specimen)	22	12.5	2489–4347	1176–2055
Wu et al., 2020 [34]	Human femoral head	9	4	314–635	1134–2293
Wu et al., 2020 [13]	3D-printed PLA/HA	20	6.5	1400–1900	1400–1900

**Table 7:** Quantification of the volumetric differences between the input model and printed parts.

Sample	Difference to Model in Total Volume (%)
Scale 4.3	6.1%
Scale 1.8	8.9%
Scale 1.5	53.6%

**Table 8:** Quantification of underfilling and overfilling for printed models of different scales (1.5, 1.8 and 4.3).

Sample	Underfilling (%) Volume Percentage of the Model that Was Not Printed	Overfilling (%) Volume Percentage of the Printed Part that Was Not in the Original Model (Extra Volume)
Scale 4.3	11	15
Scale 1.8	23	22
Scale 1.5	5	59

## Supplementary Materials

The following are available online at https://www.mdpi.com/article/10.3390/ma14133712/s1, A: Preliminary tests for the choice of printing parameters, B: Compression tests on samples with or without post-processing. Table S1. Parameters chosen for the experimental plan performed on standard cylinders printed in Black resin. The boxes highlighted in grey indicate the parameters that change from the control group; Figure S1. Elastic modulus of standard cylinders printed in Black resin, tested in compression. The errors bars represent standard deviation within the group. Statistical difference of a group is shown by *, followed by the group numbers that it is statistically significant to; Figure S2. “Cured” and “Green” parts properties comparison. The error bars represent standard deviation within the groups.
