# Supplementary material for: The Potential of Stereolithography for 3D Printing of Synthetic Trabecular Bone Structures
Source: Materials (Basel). 2021 Jul 2;14(13):3712. doi: 10.3390/ma14133712 (PMC8269906; doi:10.3390/ma14133712)
Supplement: Supplementary file 1 [file materials-14-03712-s001.zip › materials-1263071-supplementary.pdf]

materials

MDPI

Supplementary Materials

The Potential of Stereolithography for 3D Printing of Synthetic Trabecular Bone Structures

Ana Grzeszczak [1,*], Susanne Lewin [1], Olle Eriksson [2], Johan Kreuger [2] and Cecilia Persson [1]

[1] Department of Materials Science and Engineering, Uppsala University, 751 21 Uppsala, Sweden; susanne.lewin@angstrom.uu.se (S.L.); Cecilia.Persson@angstrom.uu.se (S.P.)
[2] Department of Medical Cell Biology, Uppsala University, 751 23 Uppsala, Sweden; olle.eriksson@mcb.uu.se (O.E.); johan.kreuger@mcb.uu.se (J.K.)
* Correspondence: ana.grzeszczak@angstrom.uu.se; Tel.: +46-760376722

A: Preliminary tests for the choice of printing parameters

The experimental plan described in Table S1 was carried out on standard cylinders printed in Black resin, allowing for getting a comprehensive picture of this resin and to test the processing parameters. These experiments having been conducted on dense samples, the results were compared to the properties of cortical bone, and not to those of trabecular bone. Several batches of Black resin cylinders were printed to investigate the influence of the following parameters on the mechanical properties: printer type, orientation, resolution and UV-curing time. A set of recommended parameters was chosen to establish a control batch, and only one parameter at a time was changed for each other batch. As supplementary tests, other batches were produced with additional changes in the parameter configuration, as can be seen in the lower part of Table S1. Each batch was composed of 5 samples, except for the batch "100 µm" that was composed of 2 samples. For the tests requiring a preload during the compression test, 5 preconditioning cycles were applied, with a maximum force of 900 N and a minimum force of 10 N for each cycle, performed at a crosshead speed of 1 mm/min [1].

Table S1. Parameters chosen for the experimental plan performed on standard cylinders printed in Black resin. The boxes highlighted in grey indicate the parameters that change from the control group.

Batch Name	Resolution (µm)	Orientation	Cure Time (min)	Printer	Preload during Test
Control	25	Vertical	30	Form3	No
100 µm	100	Vertical	30	Form3	No
H	25	Horizontal	30	Form3	No
60min	25	Vertical	60	Form3	No
F2	25	Vertical	30	Form2	No
Preload	25	Vertical	30	Form3	Yes
H-F2	25	Horizontal	30	Form2	No
60min-F2	25	Vertical	60	Form2	No
45min	25	Vertical	45	Form3	No

The results of the tests can be seen in Figure S1. The expected elastic modulus for the control group was 2.8 GPa (Table 2), but the test's result was significantly lower (1879 MPa with a standard deviation of 51 MPa). This elastic modulus is lower than the elastic modulus range of values for cortical bone, as described in Table 1 (12–18 GPa [2]).

The elastic modulus of the control group did not appear to be statistically different from that of the other batches, excepted for the batches of the horizontally printed samples: batch "H" and batch "H-F2". In a previous study [3], an elastic modulus of 1.33 GPa ± 0.14 GPa was obtained for samples printed with FDM in pure poly(lactic acid) (PLA), and increased with the addition of hydroxyapatite (HA) in the filament. In the present

study, the mechanical properties do not seem to be adjustable with the print settings. A significant difference was only observed when changing the print orientation of the samples. However, being able to print samples horizontally will not be the priority choice for printing trabecular samples. Indeed, with trabecular structures oriented in different directions, the initial orientation of the sample may be less important than in dense structures.

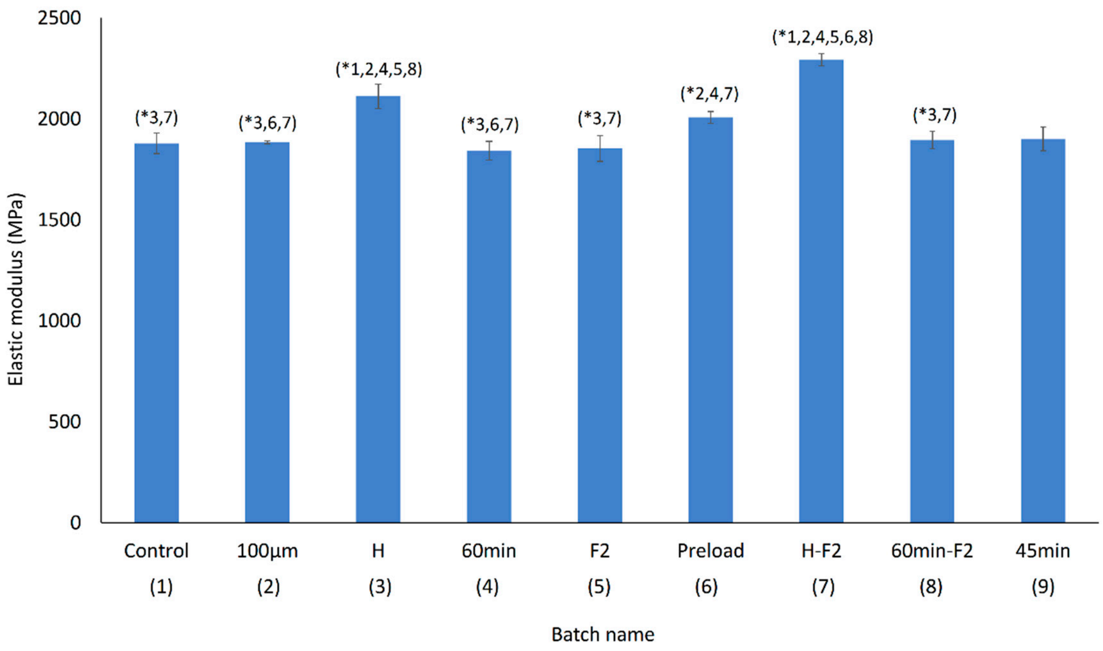

Figure S1. Elastic modulus of standard cylinders printed in Black resin, tested in compression. The errors bars represent standard deviation within the group. Statistical difference of a group is shown by *, followed by the group numbers that it is statistically significant to.

The influence of the orientation on the accuracy of the printed trabecular structures was assessed by printing samples vertically and horizontally, using the three-dimensional trabecular bone model previously mentioned with a scale factor of 4, with Black resin in a Form3 printer. The orientation of the samples on the build platform of the printer did not seem to have an influence on the ratio Bone Volume/Total Volume (BV/TV) for the samples at scale 4 (33.13% BV/TV for scale 4 vertically printed, 35.75% BV/TV for scale 4 horizontally printed).

B: Compression tests on samples with or without post-processing

To get an insight of the influence of post-treatment on SLA-printed parts, dense cylinders were printed in GreyPro and Durable resin and only half of the samples underwent UV-curing post-process. Each batch was composed of five samples. The results can be seen in Figure S2. "Green" parts refer to parts that have not been through any post-process treatment. "Cured" parts refer to parts that have been through the recommended post-process treatment (UV-light and heat exposure) for their specific type of resin. It can be noticed that the post-treatment greatly increases the stiffness of the parts. However, the elastic moduli obtained with these compression tests were lower than the expected elastic moduli, as given by the supplier Formlabs (Table 2). For the GreyPro resin, the expected elastic modulus is 2600 MPa for cured parts, and 1400 MPa for green parts. The tests results gave an elastic modulus of 1845 MPa for cured parts, with a standard deviation of 21 MPa, and an elastic modulus of 1074 MPa for green parts, with a standard deviation of 39 MPa. For the Durable resin, the expected elastic modulus is 1260 MPa for cured parts, and 450 MPa for green parts. The tests results gave an elastic modulus of 592 MPa for cured parts, with a standard deviation of 9 MPa, and an elastic modulus of 206 MPa for

green parts, with a standard deviation of 12 MPa. The results obtained for the elastic moduli did not match the expected values, they were systematically inferior. It can be mentioned that inaccuracies may have been caused by test conditions, such as a slight slippage of the samples between the test plates, or a small sample size compared to the setup used.

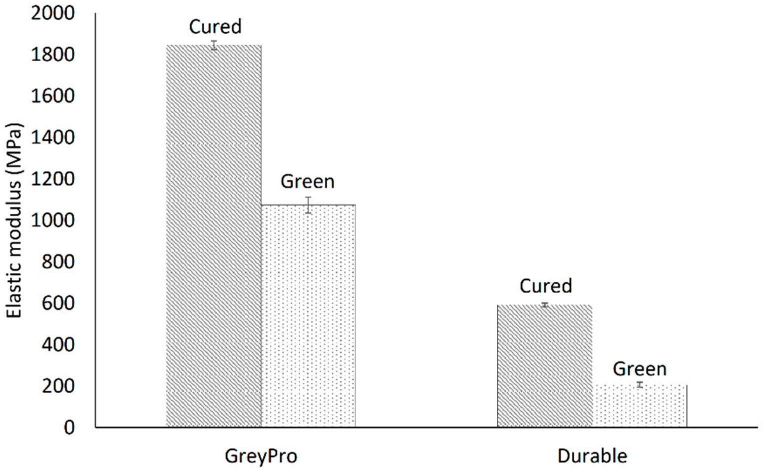

Figure S2. "Cured" and "Green" parts properties comparison. The error bars represent standard deviation within the groups.

The presence of a post-treatment with UV-light significantly influenced the elastic modulus of the material composing the parts. Although it appeared important to test the influence of post-process on mechanical properties, we must mention that we do not recommend the use of untreated samples. Indeed, their manipulation involved protecting them from light and heat and handling them with care. These constraints are not acceptable for the intended use of these materials, e.g. the fabrication of implants. In addition, the properties of untreated specimens are not stable and may vary over time. Treated specimens should be used as they are more stable.

References

1. Race, A.; Mann, K. A.; Edidin, A. A. Mechanics of bone/PMMA composite structures: An in vitro study of human vertebrae. *J. Biomech.* **2007**, *40*, 1002–1010, doi: 10.1016/j.jbiomech.2006.04.003.
2. Milazzo, M.; Contessi Negrini, N.; Scialla, S.; Marelli, B.; Farè, S.; Danti, S.; Buehler, M.J. Additive Manufacturing Approaches for Hydroxyapatite-Reinforced Composites. *Adv. Funct. Mater.* **2019**, *29*, 1903055, doi:10.1002/adfm.201903055.
3. Wu, D.; Spanou, A.; Diez-Escudero, A.; Persson, C. 3D-printed PLA/HA composite structures as synthetic trabecular bone: A feasibility study using fused deposition modeling. *J. Mech. Behav. Biomed. Mater.* **2020**, *103*, 103608, doi:10.1016/j.jmbbm.2019.103608.
